# Gestational timing and vaccine platform shape maternally derived neutralizing immunity and vaccine RNA detection in piglets following maternal vaccination with C-strain or FlagT4G against classical swine fever

**DOI:** 10.3389/fvets.2026.1827278

**Published:** 2026-05-25

**Authors:** Liani Coronado, Adriana Muñoz-Aguilera, Sara Puente-Marin, Cristina Riquelme, Saray Heredia, Iván Muñoz, Àlex Cobos, Manuel V. Borca, Llilianne Ganges

**Affiliations:** 1 WOAH Reference Laboratory for Classical Swine Fever, IRTA-CReSA, Barcelona, Spain; 2 Unitat Mixta d’Investigacio´ IRTA-UAB en Sanitat Animal, Centre de Recerca en Sanitat Animal (CReSA), Universitat Autònoma de Barcelona (UAB), Barcelona, Spain; 3 IRTA, Programa de Sanitat Animal, Centre de Recerca en Sanitat Animal (CReSA), Barcelona, Spain; 4 Subgerencia de Análisis y Diagnóstico, Instituto Colombiano Agropecuario (ICA), Bogotá, Colombia; 5 Plum Island Animal Disease Center, Agricultural Research Service, United States Department of Agriculture, Orient, NY, United States

**Keywords:** classical swine fever, C-strain, DIVA vaccine, FlagT4G, gestational vaccination, maternally derived antibodies, neutralizing antibodies

## Abstract

Control of classical swine fever (CSF) in endemic regions relies heavily on vaccination of breeding herds. Optimizing vaccination strategies is essential for sustainable disease control covering pigs belonging to different categories. Data describing direct comparisons between the classical C-strain and recombinant DIVA-compatible vaccines in the context of gestational timing, maternal antibody transfer, and early postnatal virological outcomes remain very limited. This study evaluated how vaccination in the middle to late pregnancy periods (at 72 days of gestation) with either the live attenuated C-strain or the recombinant FlagT4G vaccine influences early postnatal immunity and molecular detection profiles in piglets. Sow humoral responses induced by vaccination were assessed by E2-specific blocking ELISA and virus neutralization peroxidase-linked assay (NPLA), and piglets were monitored for the presence of maternally derived antibodies as well as vaccine-derived CSFV RNA. Vaccination at 72 days of gestation resulted in limited and transient detection of vaccine-derived RNA in piglets, without showing any clinical signs. Both vaccines induced robust E2-specific binding antibody responses in sows; however, only FlagT4G elicited high-magnitude and broadly cross-neutralizing antibody titres. Interestingly, piglets born from C-strain-vaccinated sows presented E2-specific antibodies detectable by ELISA but neutralizing activity was low and declined rapidly. In contrast, piglets derived from FlagT4G-vaccinated sows displayed stronger, more homogeneous, and functionally relevant neutralizing responses that persisted through the early neonatal period. Notably, following vaccination at 72 days of gestation, the gradual decline of maternally derived neutralizing titres coincided with the typical weaning age (approximately 28–35 days), potentially facilitating timely piglet vaccination with reduced interference. In a second experiment it is shown that vaccination at 44 days of gestation with FlagT4G consistently induced high neutralizing titres in sows and a highly efficient transfer of functional maternally derived immunity to piglets, without detectable vaccine RNA at birth. Overall, gestational timing and vaccine platform critically influenced the magnitude and functional quality of maternally derived immunity. Reliance solely on ELISA-based E2 antibody detection may overestimate the presence of protective immunity, emphasizing that the quantitative and functional assessment of neutralizing responses is essential to optimize maternal immunization strategies and advance sustainable DIVA-compatible CSF control.

## 1 Introduction

Classical swine fever (CSF) is a highly contagious viral disease of domestic pigs and wild boar caused by classical swine fever virus (CSFV), a member of the genus *Pestivirus* within the family *Flaviviridae* (1, 2). The disease is characterized by a broad clinical spectrum ranging from acute hemorrhagic fever with high mortality to subacute, chronic, or subclinical infections, depending on viral virulence, host age, immune status, and epidemiological context (1, 3). Although CSF has been successfully eradicated from several regions through coordinated control programs, it continues to circulate in parts of Asia, Latin America, and Eastern Europe, where it represents a persistent threat to pig production, food security, and international trade (1, 4).

Vaccination remains a central pillar of CSF control strategies in endemic areas and in emergency response situations. Among available vaccines, the live attenuated C-strain, derived from the Chinese “hog cholera lapinized virus”, has been extensively used for decades due to its strong immunogenicity, rapid onset of protection, and favorable safety profile (1, 5). Experimental studies have shown that C-strain vaccination can confer protection as early as 5–7 days post-immunization, even against heterologous CSFV genotypes (6). Despite these advantages, a major limitation of the classical C-strain vaccine is its inability to differentiate infected from vaccinated animals (DIVA), which complicates surveillance and trade in regions aiming for disease-free status (1, 4). To address these limitations, recombinant marker vaccines have been developed that combine protective efficacy with DIVA compatibility. Among these, the recombinant FlagT4G vaccine represents a promising next-generation live attenuated candidate. Previous studies have demonstrated that FlagT4G induces rapid and robust protective immunity following a single administration, exhibits genetic stability, and allows serological differentiation between infected and vaccinated animals (7–9). Importantly, recent work has shown that vaccination of pregnant sows with FlagT4G can prevent transplacental transmission of highly virulent CSFV strains, even when vaccination is performed during mid-gestation and relatively close to viral challenge (10, 11). These findings highlight the potential of FlagT4G as a suitable candidate for use in breeding herds under endemic conditions.

Beyond direct protection of vaccinated animals, an essential yet comparatively underexplored component of CSF vaccination programs is the role of maternally derived antibodies (MDA) in piglet protection. Piglets are born agammaglobulinemic due to the epitheliochorial structure of the porcine placenta and depend entirely on colostrum intake for passive transfer of immunoglobulins (12). The magnitude and persistence of maternally derived antibodies in piglets are influenced by multiple factors, including the immune status of the sow, the vaccine used, the timing of vaccination during gestation, and the adequacy of colostrum intake after birth. Sufficient ingestion of high-quality colostrum is critical for the effective transfer of passive immunity to neonatal piglets. Effective maternal immunization can therefore play a decisive role in protecting piglets during the early postnatal period, when they are particularly susceptible to infection. However, maternally derived antibodies represent a double-edged sword. While they provide essential early-life protection, high antibody titres at the time of piglet vaccination may interfere with the induction of active immunity. Experimental studies have demonstrated that elevated maternal antibody levels can suppress both humoral and cellular immune responses to CSF vaccination (12, 13). More recent evaluations have reinforced the concept that maternal antibody interference is quantitative and time-dependent, with vaccine efficacy depending on antibody magnitude at the time of piglet immunization (14). Additionally, challenge studies have described a narrow window of protection mediated by maternally derived antibodies, in which intermediate antibody levels may confer protection against virulent CSFV, whereas low titres fail to protect and very high titres may interfere with vaccine-induced immune responses (15). These findings underscore the importance of understanding maternal antibody kinetics when designing vaccination schedules.

Despite the long-standing use of the C-strain vaccine and the growing interest in recombinant marker vaccines such as FlagT4G, there remains limited information on how vaccination at different gestational stages influences maternal antibody transfer, early innate immune responses, and molecular virological profiles in piglets. In particular, comparative data between CSF vaccines in the context of gestational timing are scarce.

Furthermore, while the safety and efficacy of FlagT4G in pregnant sows have been demonstrated under challenge conditions (10, 11), the early postnatal clinical and virological profiles of piglets born to vaccinated sows have not been comprehensively characterized under standardized gestational vaccination protocols for either vaccine platform. In addition, the potential detection of vaccine-derived CSFV RNA in piglets following gestational vaccination has not been systematically evaluated for both vaccines. In the context of increasing reliance on molecular diagnostic tools, understanding whether limited and transient detection of vaccine-derived RNA may occur in clinically healthy piglets is essential for accurate interpretation of surveillance data and for reinforcing confidence in the safety of gestational vaccination strategies.

Despite being a globally prioritized swine disease, CSF vaccination coverage reached only 6.56% of at-risk pig populations in 2025, with marked regional variation (16). Although several countries have achieved CSF-free status, endemic areas continue to rely on large-scale vaccination programs that must balance protective efficacy with surveillance requirements. In this context, the evaluation of DIVA-compatible vaccines and the immunological consequences of gestational vaccination are critical to refine sustainable control strategies.

Therefore, the objective of this study was to comprehensively evaluate how the timing of gestational vaccination in sows, using either the classical live attenuated C-strain vaccine or the recombinant marker FlagT4G vaccine, influences early postnatal outcomes in their offspring. Specifically, we aimed to characterize (i) the magnitude and kinetics of maternally derived humoral immunity, including E2-specific and neutralizing antibodies; and (ii) the presence, distribution, and magnitude of vaccine-derived CSFV RNA in serum, swabs, and selected tissues during the neonatal period. By integrating virological, immunological, and clinical parameters across distinct gestational vaccination time points, this study sought to determine whether vaccination timing differentially shapes early-life immune landscapes and molecular detection profiles in piglets. A deeper understanding of these dynamics is essential to optimize maternal immunization strategies, balance early passive protection with potential interference in piglet vaccination and further substantiate the safety and field applicability of recombinant DIVA-compatible vaccines such as FlagT4G in breeding herds.

## 2 Materials and methods

### 2.1 Cells and viruses

The Pestivirus-free porcine kidney cell line PK-15 (ATCC CCL-33) was maintained in Eagle’s minimum essential medium (EMEM) supplemented with 5% fetal calf serum, 1% non-essential amino acids, and antibiotics, under standard culture conditions (37 °C, 5% CO₂). This cell line was used for virus propagation, titration, and neutralization assays as previously described for CSFV experimental systems (10, 17). Viral titers were determined by end-point dilution assay using a peroxidase-linked detection method (PLA) as previously described (18). Infectious titers were calculated using the Reed and Muench method (19) and expressed as 50% tissue culture infectious doses (TCID₅₀/mL). The recombinant live attenuated FlagT4G DIVA vaccine virus, developed and characterized as explained in previous reports, was used for *in vivo* vaccination experiments (7). The live attenuated CSFV vaccine based on the C-strain (Pestiffa strain). The CSFV reference strains Alfort/187 and Diepholz 1, kindly provided by the European Union Reference Laboratory for CSF (Hanover, Germany), were used for neutralization assays to assess functional antibody responses, as previously described (10, 11).

### 2.2 Animals and experimental design

In this study, six Pestivirus-free Landrace sows were obtained from a commercial farm with no prior history of CSFV infection or vaccination. Animals were housed under biosafety level 3 (BSL-3) containment conditions at the IRTA-CReSA facilities (Barcelona, Spain) for the duration of the experimental procedures. All animals were confirmed negative for Pestivirus antibodies and viral RNA prior to inclusion. The study comprised two independent experimental settings designed to evaluate the impact of gestational vaccination timing. The vaccination dose, route of administration, and handling were consistent with previously validated vaccination protocols (10, 11) and aligned with recommendations described in the WOAH Manual (4). Post-vaccination monitoring included daily clinical assessment performed by a trained veterinarian blinded to vaccine allocation, following previously described methodologies (9, 20).

#### 2.2.1 Vaccination at 72 days of gestation

Four pregnant sows were enrolled in the first experimental setting. Animals were acclimatized for one week under controlled environmental conditions before vaccination. At 72 days of gestation, sows were randomly allocated to two vaccination groups, using two animals per groups (21, 22). The FlagT4G vaccine included sow numbered 1 and 2 and the live attenuated C-strain vaccine group, sows numbered 3 and 4. The FlagT4G group was intramuscularly vaccinated in the neck with 10^5^ TCID₅₀ per animal (11), while the C-strain group was vaccinated intramuscularly with 2 mL per dose (minimum potency 100 PD₅₀), corresponding to the manufacturer-recommended protective dose validated in swine. Both vaccination regimens comply with established potency standards. The C-strain dose used here is consistent with previously published experimental studies in pigs (23).

**Figure 1:**
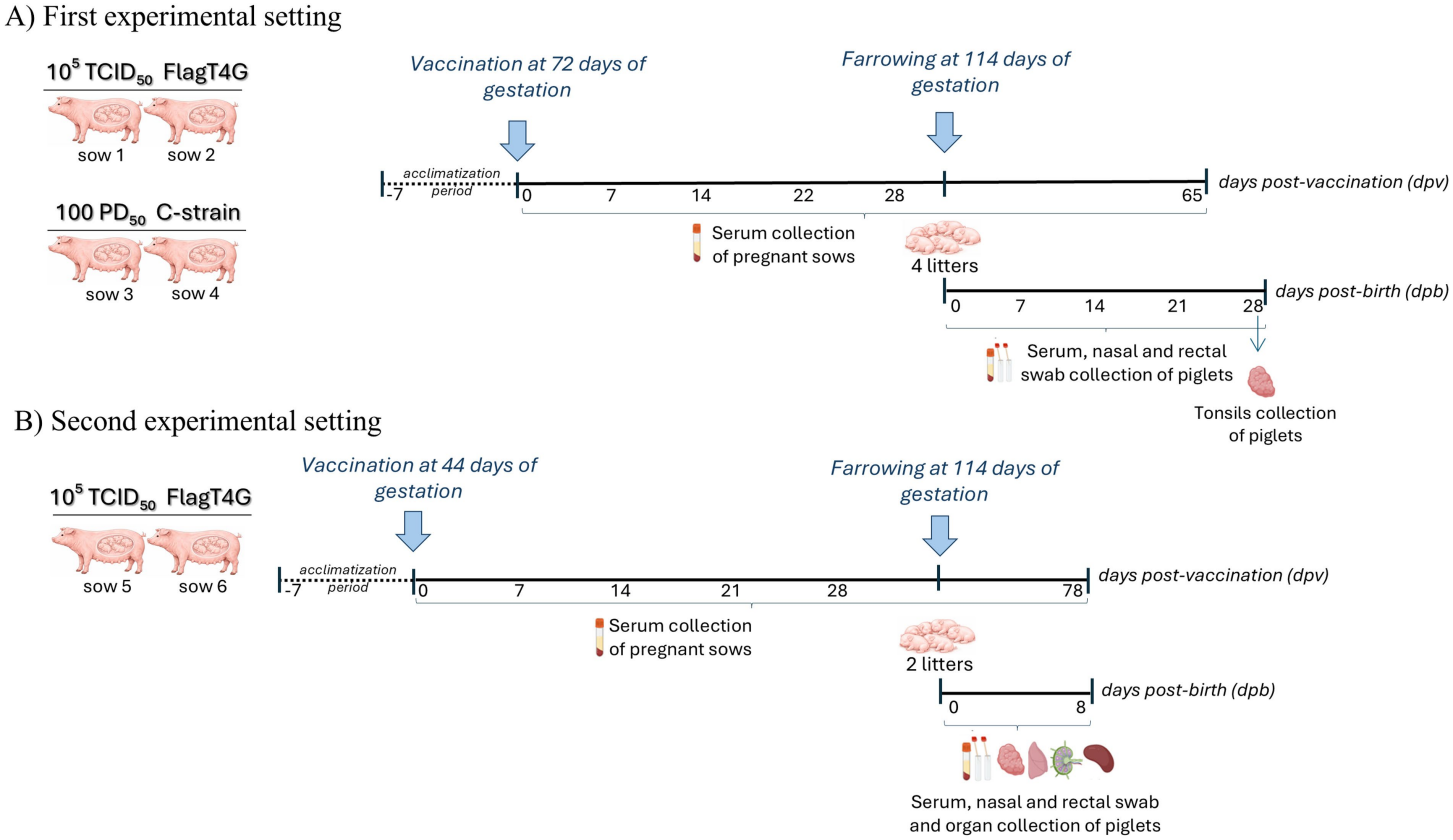
Experimental design of vaccinated pregnant sows. **(A)** First experimental setting: four sows were vaccinated at 72 days of gestation, two with FlagT4G and two with the C-strain vaccine. **(B)** Second experimental setting: two pregnant sows were vaccinated with FlagT4G at 44 days of gestation. In both experimental settings, farrowing occurred at 114 days of gestation.

Parameters recorded included rectal temperature, appetite, behavior, and general health condition. Serum samples were collected from vaccinated sows at the day of vaccination [0 days post-vaccination, (dpv)] and at 7, 14, 22, 28 and 65 dpv, following standardized CSFV sampling procedures (10, 11). Farrowing occurred at approximately 114 days of gestation. Immediately after parturition, both sows and piglets were clinically evaluated. During the postpartum follow-up, sows and their litters were monitored daily for clinical signs and piglets were sampled weekly until 28 days post-birth (dpb) (7, 14, 21, and 28 dpb). At each time point, serum samples were collected for both viral RNA detection and serological analyses and nasal and rectal swabs were obtained to assess viral RNA detection dynamics. At the end of the follow-up period, 28 dpb for piglets and 65 dpv for sows, they were humanely euthanized. Piglets were subjected to complete necropsy and tissue samples, including tonsils, were collected following the same standardized necropsy protocol described above (Figure 1A).

#### 2.2.2 Vaccination at 44 days of gestation with FlagT4G and neonatal characterization

Two pregnant sows (numbered 5 and 6) were included in a second independent experimental setting designed to evaluate the safety of early gestational vaccination with the recombinant FlagT4G vaccine and to characterize the immediate postnatal clinical, virological, and immunological profile of their offspring. Following a one-week acclimatization period, sows were vaccinated intramuscularly at 44 days of gestation with 10^5^ TCID₅₀ of the recombinant FlagT4G vaccine per animal. Clinical monitoring included rectal temperature, appetite, behavior, and general health status. Serum samples were collected at 0, 7, 14, 21, 28 and 78 dpv. Farrowing occurred at approximately 114 days of gestation. Immediately after parturition (0 dpb), all piglets were individually identified and subjected to thorough clinical examination to assess vitality, congenital abnormalities, and overall health status. Postpartum clinical monitoring of both sows and piglets was conducted daily throughout the observation period (Figure 1B).

At birth (0 dpb), half of the piglets from each litter were randomly selected for serum, nasal and rectal swabs collection to evaluate vaccine-derived CSFV RNA and maternally derived antibody responses. Then, these piglets, half from each litter, were humanely euthanized at 0 dpb for detailed tissue-based analysis. Tissue samples were collected from tonsil, spleen, thymus, lung, and mesenteric lymph node. The remaining piglets were maintained under BSL-3 containment conditions and resampled at 8 dpb, when serum, nasal swabs, and rectal swabs were again collected to assess early postnatal dynamics of viral RNA detection and maternally derived immunity. At this time point, these piglets were humanely euthanized and subjected to complete necropsy, and tissue samples were collected following the same standardized protocol described above.

#### 2.2.3 Ethical approval and animal welfare compliance

All animal procedures were conducted in accordance with Directive 2010/63/EU on the protection of animals used for scientific purposes and were approved by the Animal Experimentation Committee of the Generalitat of Catalonia (authorization number 10907), on 3 September 2020. Experimental procedures were performed following WOAH guidelines for CSFV studies (4).

### 2.3 Detection of CSFV RNA

Viral RNA was extracted from serum, nasal and rectal swabs, and homogenized tissue samples using the IndiMag® Pathogen Kit (Indical Bioscience, Leipzig, Germany), following the manufacturer’s instructions. Tissue samples were homogenized in EMEM (1 g tissue + 9 mL medium) supplemented with antibiotics prior to extraction. Quantitative reverse transcription PCR (RT-qPCR) targeting the CSFV 5′ untranslated region (5′UTR) was performed using validated protocols for CSFV molecular detection (4, 24). Samples with Ct values ≤ 40 were considered positive, in accordance with established diagnostic thresholds. Ct values were recorded to estimate relative viral RNA load.

### 2.4 Determination of E2-specific antibodies

E2-specific antibodies in sow and piglet sera were quantified using a commercial blocking ELISA kit (IDEXX Laboratories, Liebefeld, Switzerland), following the manufacturer’s instructions. Blocking percentages ≥40% were considered positive, consistent with validated cut-off values (1, 10).

### 2.5 Neutralization peroxidase-linked assay (NPLA)

Neutralizing antibody titers against CSFV strains Alfort/187 and Diepholz 1 and FlagT4G vaccine were determined using a neutralization peroxidase-linked assay (NPLA), as previously described for CSFV seroneutralization (25). Briefly, serial two-fold dilutions of each heat-inactivated serum sample was incubated with 100 TCID₅₀ of each virus for 1 h at 37 °C, before inoculation onto PK-15 cell monolayers. After incubation period of 72 h at 37 °C, infected cells were detected by peroxidase staining. Neutralizing titers were expressed as the reciprocal of the highest serum dilution capable of neutralizing specific viral strain in 50% of replicate wells.

## 3 Results

### 3.1 Clinical outcome in sows and piglets with vaccination performed at 72 days of gestation

No adverse clinical signs were observed in any sow following vaccination with either the C-strain or the FlagT4G vaccine. All vaccinated sows remained clinically healthy throughout gestation and until farrowing. Sow 1 delivered a total of 23 piglets, including 4 stillborn and 3 mummified, resulting in 16 live-born piglets. Sow 2 delivered 20 piglets, of which 2 were stillborn; an additional 2 piglets were euthanized at birth to standardize litter size, resulting in 16 piglets. Sow 3 delivered 16 piglets, including 2 mummified and 1 stillborn, resulting in 13 live-born piglets. Sow 4 delivered 14 piglets, including 2 stillborn and 1 mummified; one additional piglet died shortly after birth due to crushing by the sow, resulting in 10 piglets. During the first 2 weeks post-farrowing, litter sizes were adjusted to optimize teat allocation and ensure adequate nursing conditions. Consequently, at 7 dpb, 14 piglets remained in litter 1, 16 in litter 2, 13 in litter 3, and 10 in litter 4 and at 14 dpb, 13 piglets remained in litter 1, 15 in litter 2, 12 in litter 3 and 10 in litter 4. As part of this standardized management procedure, a subset of piglets from each litter was humanely euthanized at 21 dpb due to reduced body condition associated with limited milk availability in specific litters. Therefore, at 21 and 28 dpb, the remaining animals (11 in litter 1, 14 in litter 2, 9 in litter 3, and 10 in litter 4) were subsequently followed longitudinally according to the experimental protocol. Importantly, no clinical signs or lesions compatible with CSFV were observed in any piglet at birth or throughout the entire postnatal observation period.

### 3.2 Detection of CSFV RNA in piglets born to sows vaccinated at 72 days of gestation

**Figure 2:**
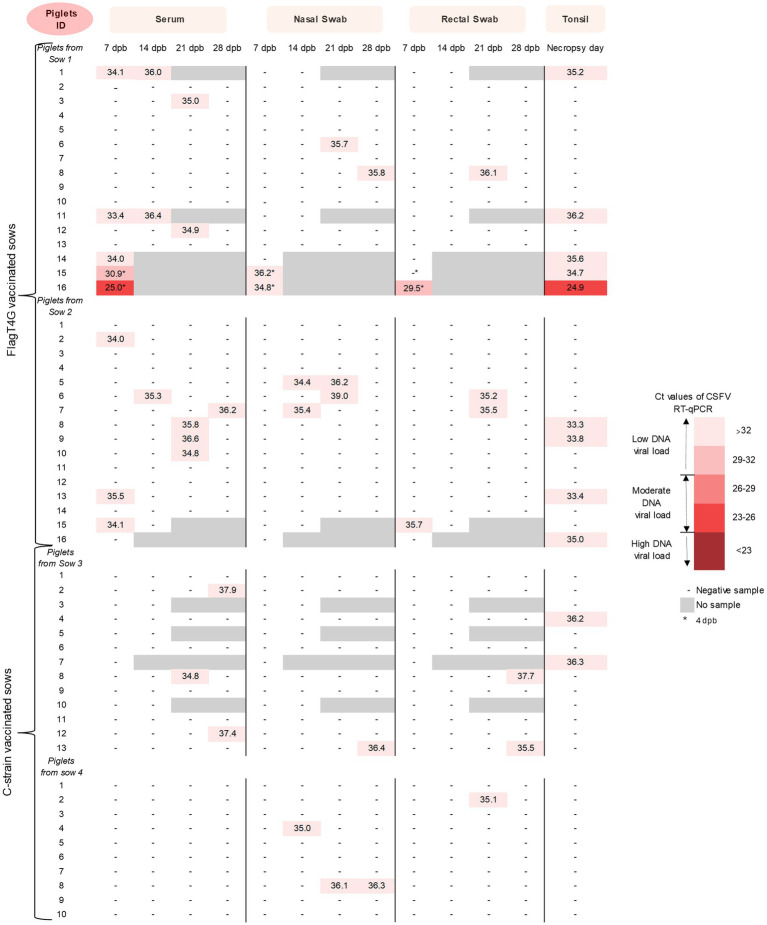
Detection of CSFV RNA by RT-qPCR in serum, swab, and tonsil samples from piglets born to sows vaccinated at 72 days of gestation with FlagT4G or the C-strain. Viral RNA load, expressed as Ct values, is represented as low, moderate, or high according to the intensity of the red color scale.

In this experimental setting sampling of piglets began at 7 dpb, and the distribution of positive samples and corresponding Ct values are shown in Figure 2. In piglets born to C-strain-vaccinated sows, positive results were limited to isolated animals and sample types. Detected Ct values were predominantly above 34, indicating low RNA levels. Serum, nasal, and rectal swabs showed occasional positivity, without consistent detection across multiple compartments within the same animal (Figure 2). No progressive increase in detection frequency was observed at later time points. In piglets derived from FlagT4G-vaccinated sows, RT-qPCR positivity was more frequently observed than in the C-strain group; however, detection remained scattered and heterogeneous. Most Ct values were above 32, consistent with low RNA loads. A single sample (piglet #16 at 7 dpb) exhibited a Ct value of 25, corresponding to a moderate RNA signal. Importantly, this finding was restricted to one compartment at a single time point and was not accompanied by clinical signs or pathological lesions. During subsequent sampling points (14–28 dpb), detection remained intermittent across samples. Tonsil samples collected at necropsy occasionally yielded positive results, again characterized by high Ct values (>34) in most cases (Figure 2).

Overall, in piglets born to sows vaccinated at 72 days of gestation, vaccine-derived CSFV RNA detection was limited, transient, and predominantly associated with low-level RNA signals. Positive RT-qPCR results were sporadic and not indicative of sustained or systemic viral dissemination. No clinical signs compatible with CSF were observed in any RT-qPCR–positive animal.

### 3.3 Kinetics of E2-specific and neutralizing antibody responses in sows vaccinated at 72 days of gestation: C-strain versus FlagT4G

**Figure 3:**
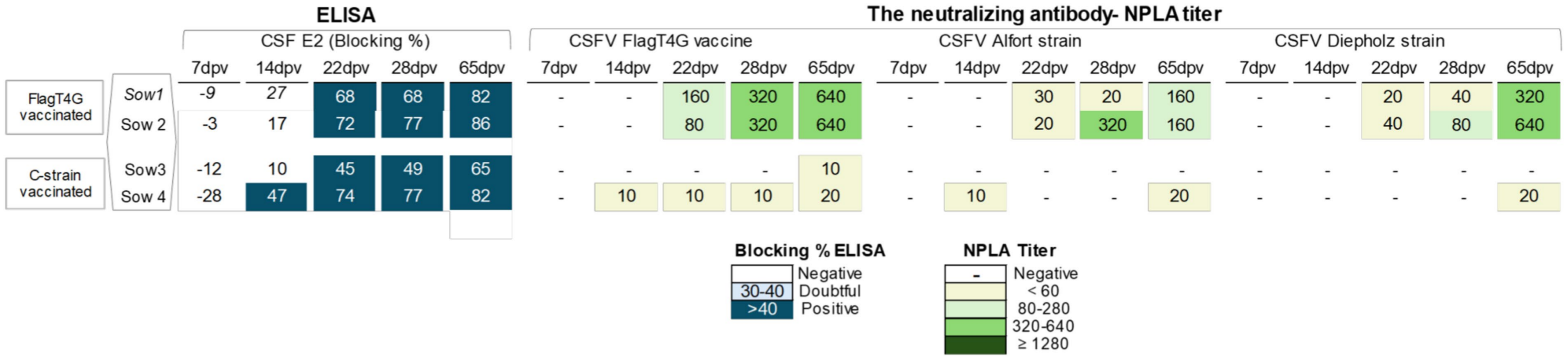
Humoral immune response in sows vaccinated at 72 days of gestation with FlagT4G or the C-strain. CSFV E2-specific antibodies were measured by blocking ELISA, and neutralizing antibody titers were determined by NPLA. Results are shown for each individual animal at different time points post-vaccination. ELISA blocking values are expressed as percentages and are color-coded: negative (−), doubtful (30–40%), or positive (>40%). Neutralizing titers are presented numerically and represented using a graded green color scale ranging from light (low titers) to dark (high titers). Hyphens indicate absence of detectable neutralizing activity.

E2-specific antibodies were quantified by blocking ELISA and expressed as blocking percentages (Figure 3). Following vaccination at 72 days of gestation, all sows were ELISA-negative at 7 dpv, with blocking values below the 40% positivity threshold. At 14 dpv, early seroconversion was evident in one C-strain-vaccinated sow (47%), while both FlagT4G-vaccinated sows were still ELISA-negative at this time point. By 22 dpv, all animals in both groups had seroconverted, with blocking percentages exceeding 40%. From this time point onward, E2-specific responses remained consistently high through 28 dpv and 65 dpv (23 days post-farrowing; lactation period) with blocking values ranging approximately between 65 and 86%. The magnitude of ELISA-detectable antibodies was comparable between vaccine groups during late gestation and early lactation (Figure 3), indicating sustained CSFV E2 glycoprotein-specific humoral immunity.

In contrast, functional neutralising responses differed markedly between vaccines. C-strain-vaccinated sows generated low or undetectable neutralising titres against the tested viruses at 22 dpv, with titres typically ranging between negative and between 10 and 20. Only minimal increases were observed by 28 and 65 dpv, and neutralising activity against heterologous strains remained low (Figure 3). FlagT4G vaccination, however, induced substantially higher neutralising titres as early as 22 dpv, with titres reaching 160 to 320 against homologous virus and measurable cross-neutralisation against Alfort (CSFV genotype 1.1) and CSFV Diepholz 1 (genotype 2.3). By 28 dpv and 65 dpv, neutralising titres further increased, reaching values up to 640 (Figure 3). Thus, while both vaccines elicited robust E2-specific binding antibody responses detectable by ELISA, only FlagT4G induced high-magnitude and broadly cross-reactive neutralising antibody responses across multiple CSFV strains.

### 3.4 . Maternally derived E2-specific antibodies in piglets born to sows vaccinated at 72 days of gestation

**Figure 4:**
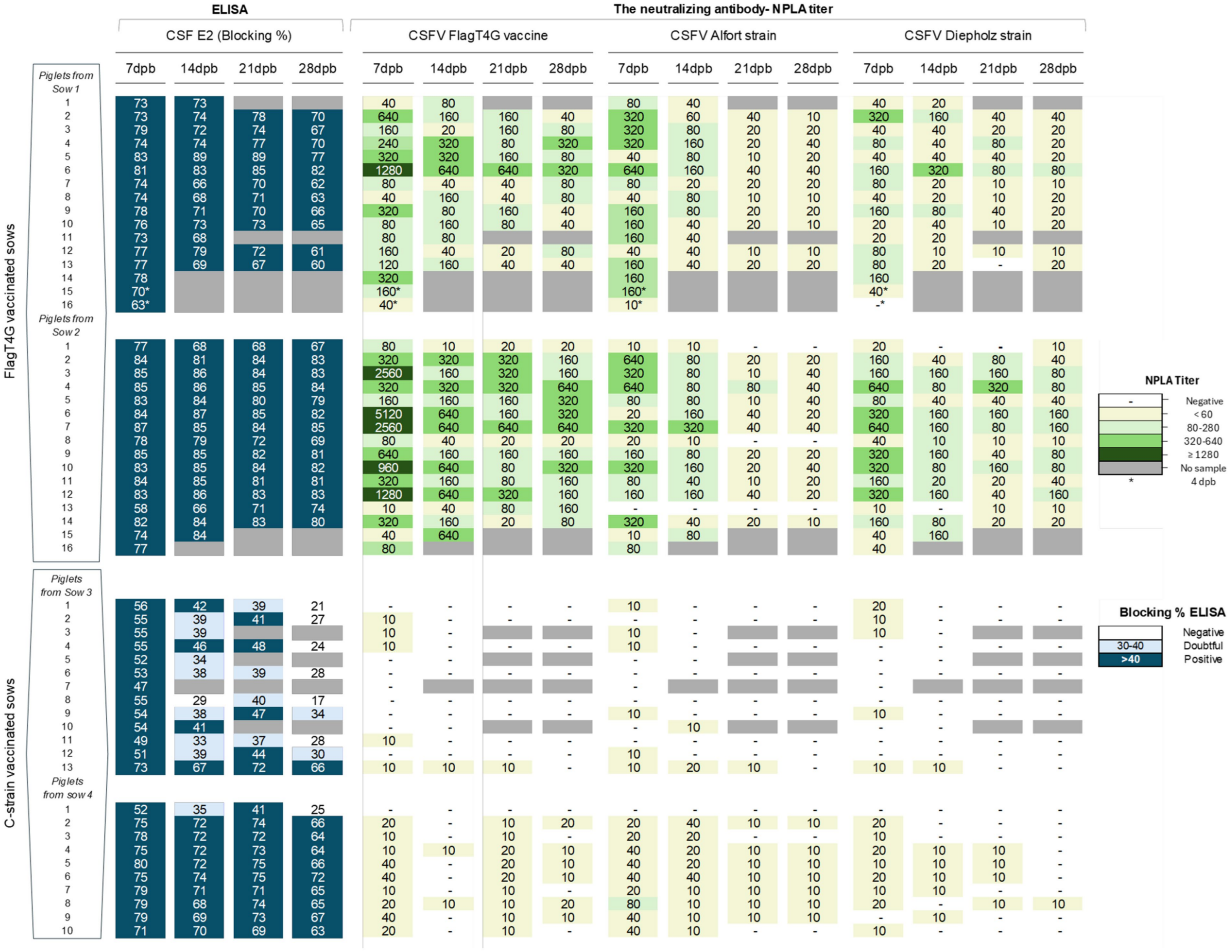
Humoral immune response in piglets born to sows vaccinated at 72 days of gestation with FlagT4G or the C-strain. CSFV E2-specific antibodies measured by blocking ELISA and neutralizing antibody titers determined by NPLA. Results are shown for each individual animal. ELISA blocking values are expressed as percentages and are color-coded: negative (<30%), doubtful (30–40%), or positive (>40%). Neutralizing titers are presented numerically and represented on a graded green color scale from light (low titers) to dark (high titers). Hyphens indicate absence of detectable neutralizing activity. Grey cells indicate unavailable sampling time points due to additional piglets were humanely euthanized.

In piglets, maternally derived E2-specific antibodies were detectable from 7 days post-birth (dpb) onwards (Figure 4). At 7 dpb, all evaluated piglets in both experimental groups were ELISA-positive (blocking ≥40%). Piglets born to C-strain-vaccinated sows showed blocking percentages ranging approximately from 47 to 80%, whereas piglets derived from FlagT4G-vaccinated sows exhibited values ranging from 58 to 89% (Figure 4). At 14 dpb, a clear divergence between groups was observed. In piglets born to C-strain-vaccinated sows, E2-specific blocking values declined markedly in one litter, where eight piglets became ELISA-negative (<40%) and only four remained above the positivity threshold. In the second C-strain litter, only one piglet fell below 40%, while the others remained positive. In contrast, all piglets derived from FlagT4G-vaccinated sows remained ELISA-positive at 14 dpb. By 28 dpb, the downward trend continued in C-strain group, especially in piglets from sow 3 in which a substantial proportion of piglets exhibited blocking values close to or below the ELISA cutoff, indicating progressive loss of detectable maternally derived E2-specific antibodies. Notably, in the FlagT4G group, blocking percentages stayed above the 40% threshold in all evaluated animals from 7 to 28 dpb; variations over time reflected inter-individual differences or sampling gaps rather than loss of seropositivity (Figure 4).

### 3.5 Neutralising antibody responses in piglets born to sows vaccinated at 72 days of gestation

In line with the ELISA findings, marked differences were also observed in the functional quality of maternally derived antibodies as assessed by virus neutralization (Figure 4). In piglets born to C-strain-vaccinated sows, neutralizing activity was limited. At 7 dpb, only three piglets from sow 4 reached titres of 1:40 against the homologous C-strain virus, while six animals showed titres of 1:40 against the heterologous Alfort strain. A pronounced decline in neutralizing titres was evident by 14 dpb, with virtually no piglets maintaining titres ≥1:40 against any of the tested strains. From this time onward, most titres in the C-strain group fell below the 1:40 threshold, indicating rapid decay of functional neutralizing activity despite the presence of detectable binding antibodies by ELISA. In contrast, piglets born to FlagT4G-vaccinated sows exhibited a markedly stronger and more homogeneous neutralizing antibody profile (Figure 4). At 7 dpb, virtually all piglets from both litters, except for a single individual, displayed neutralizing titres ≥1:40 across the tested strains. Titres were consistently elevated, indicating robust and broadly reactive maternally derived functional immunity. Although a gradual physiological decline was observed over time, most piglets maintained neutralizing titres at or above the 1:40 threshold through 14, 21, and in many cases 28 dpb, particularly against homologous and closely related strains (Figure 4).

### 3.6 Clinical status and CSFV RNA detection in piglets born to sows vaccinated with FlagT4G at 44 days of gestation

**Figure 5:**
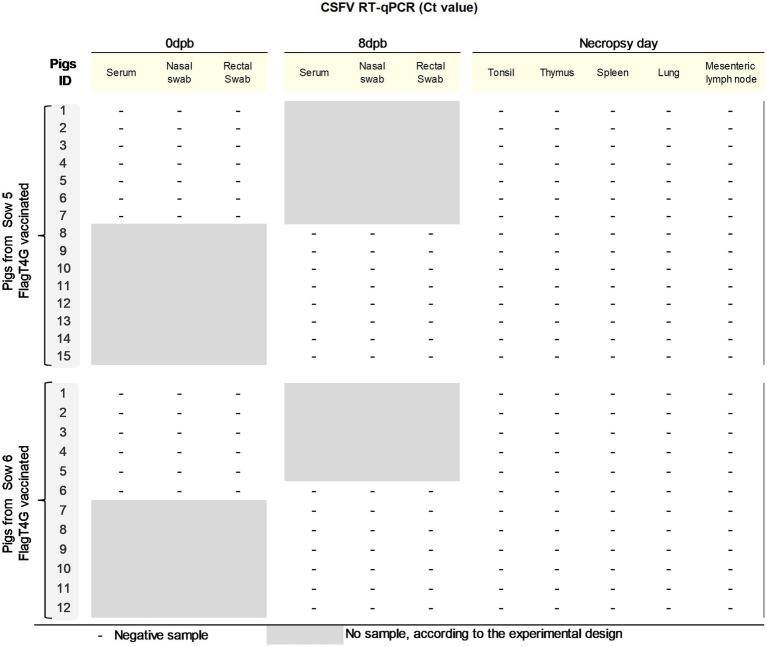
Detection of CSFV RNA by RT-qPCR in serum, swab, and tissue samples from piglets born to sows vaccinated at 44 days of gestation with FlagT4G.

Sow 5 delivered a total of 15 piglets, including 2 stillborn, resulting in 13 live-born piglets. Sow 6 delivered 13 piglets, including 1 stillborn, resulting in 12 live-born piglets. No macroscopic lesions consistent with CSF were observed in the stillborn piglets. All live-born piglets were clinically normal at birth and remained healthy throughout the observation period, with no clinical signs compatible with CSF. RT-qPCR analysis did not detect CSFV RNA at any evaluated time point in samples collected from piglets born to sows vaccinated at 44 days of gestation. No Ct values within the positivity threshold were recorded in any piglet during the study (Figure 5).

### 3.7 Kinetics of sow humoral immunity and maternally derived antibodies following vaccination at 44 days of gestation

**Figure 6:**
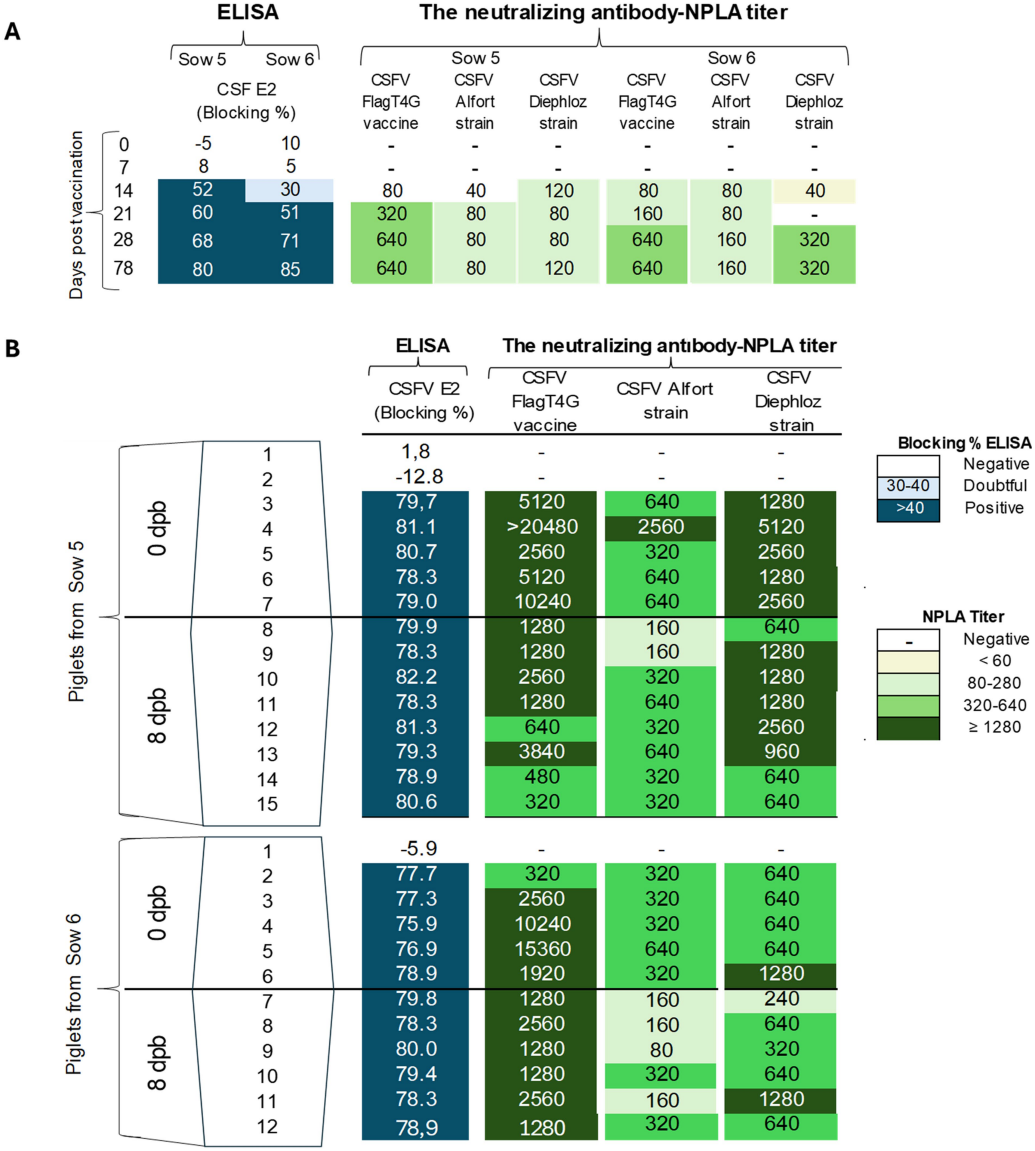
Humoral immune response in sows vaccinated at 44 days of gestation with FlagT4G **(A)** and in their piglets **(B)**. CSFV E2-specific antibodies were measured by blocking ELISA and neutralizing antibody titers were determined by NPLA. Results are shown for each individual animal. ELISA blocking values are expressed as percentages and are color-coded: negative (<30%), doubtful (30–40%), or positive (>40%). Neutralizing titers are displayed numerically and represented on a graded green color scale ranging from light (low titres) to dark (high titres) intensity. Hyphens indicate absence of detectable neutralizing activity.

Sows vaccinated with FlagT4G at 44 days of gestation developed a rapid and robust E2-specific antibody response as measured by blocking ELISA. Seroconversion was evident by 14 days post-vaccination (dpv), with blocking percentages exceeding the assay positivity threshold (≥40%). From 22 dpv onwards, both animals showed high blocking values that remained consistently elevated through 28 dpv and up to 65 dpv (corresponding to 23 days post-birth), indicating sustained humoral immunity throughout late gestation and early lactation (Figure 6A).

Neutralising antibody responses assessed by NPLA confirmed the functional quality of this humoral response. By 22 dpv, high neutralising titres were detected against the CSFV strains tested. Titres further increased at 28 dpv and remained elevated at 65 dpv, demonstrating broad cross-neutralising capacity prior to farrowing (Figure 6A). These data indicate that vaccination at 44 days of gestation induced not only strong E2-binding antibodies but also high-magnitude functional neutralising immunity in the sows.

In piglets born to these sows, maternally derived E2-specific antibodies were readily detectable by ELISA. At birth (0 dpb), most piglets exhibited very high blocking percentages, frequently exceeding 75% and approaching assay saturation levels. The only ELISA-negative animals were three piglets (identified as numbers 1 and 2 from sow 5, and number 1 from sow 6), which were stillborn and therefore had not ingested colostrum (Figure 6B).

By 7–8 dpb, E2-specific antibodies remained strongly detectable in all evaluated piglets. Blocking percentages were consistently high and well above the ≥40% threshold, indicating sustained and homogeneous maternally derived antibody levels during the first week of life (Figure 6B).

Neutralising antibodies were also readily detectable at birth in nearly all piglets. Several animals displayed titres at or above the ≥1:40 reference threshold, with some piglets reaching exceptionally high values exceeding 1:20.480, confirming highly efficient transfer of functional maternally derived immunity. At 7–8 dpb, neutralising activity remained detectable in most piglets. Although some inter-individual variability was observed and titres declined in certain animals compared with birth values, neutralising responses generally remained elevated during the first week of life, with most piglets maintaining titres within or above ranges associated with experimental protection (Figure 6B). Overall, vaccination at 44 days of gestation resulted in strong systemic humoral responses in sows and highly efficient transfer of both E2-binding and functional neutralising antibodies to piglets during the early neonatal period.

## 4 Discussion

Despite the widespread use of live attenuated vaccines for CSF control, the development and implementation of DIVA-compatible vaccination strategies remain a central objective in regions aiming to reconcile effective disease control with surveillance transparency and trade continuity (1, 4, 26). Classical C-strain vaccines have demonstrated robust protective efficacy for decades; however, their inability to serologically differentiate infected from vaccinated animals limits their applicability in eradication-oriented programs (26). In this context, recombinant DIVA-compatible platforms such as FlagT4G represent a strategic advancement, combining attenuation, genetic stability, and diagnostic compatibility (7, 9, 10). Nevertheless, information on how gestational use of DIVA vaccines shapes early postnatal immunity and CSFV RNA detection profiles in offspring remains limited.

This study demonstrates that the timing of sow vaccination with live attenuated CSFV vaccines, including the C-strain and the recombinant FlagT4G vaccine, modulates early vaccine virus RNA detection and the profile of maternally derived immunity in piglets, without inducing clinical disease. Our results provide novel insight into how gestational timing shapes early postnatal virological and immunological outcomes. A key finding of this work is the timing-dependent pattern of vaccine-derived CSFV RNA detection in piglets. Overall, RNA detection was low and transient in both 72-day vaccination groups, with RT-qPCR positivity mainly reflecting low-level vaccine-derived RNA in serum, nasal and rectal swabs, and occasionally tonsils, without evidence of systemic replication or clinical disease. In contrast, no vaccine-derived RNA was detected at birth following vaccination at 44 days of gestation.

Notably, this limited RNA detection occurred in piglets from both FlagT4G- and C-strain-vaccinated sows, despite the lower commercial dose of the C-strain vaccine compared with the experimental FlagT4G dose used in this study. It should be emphasized that the FlagT4G dose employed here reflects an experimental setting and does not represent the final field dose, as ongoing dose-optimization studies are being conducted to define the minimal effective protective dose under production conditions (27). Collectively, these findings support the interpretation that the detected RNA reflects limited perinatal exposure or residual vaccine traces rather than uncontrolled replication or pathogenic transmission. Nevertheless, this information is of considerable diagnostic relevance in countries where vaccination is implemented, as highly sensitive molecular assays may detect low-level vaccine-derived RNA in clinically healthy animals, requiring careful interpretation within vaccinated populations. Vaccination performed closer to parturition than the 72-day gestational time point evaluated here may increase the likelihood of transient perinatal vaccine-derived RNA detection in offspring due to the shorter interval between immunization and farrowing. Although this requires formal investigation, such scenarios could further complicate molecular diagnostic interpretation of CSF in vaccinated herds and therefore warrant additional study.

Notably, all piglets were born clinically healthy, regardless of vaccination timing or vaccine type. The absence of clinical signs, even in piglets with detectable vaccine virus RNA, supports the safety of both vaccines when administered during gestation and is consistent with previous studies demonstrating the attenuation and safety profile of live CSFV vaccines, including FlagT4G (7, 10, 11, 28). Our data extend these observations by demonstrating that limited vaccine RNA detection in piglets does not translate into adverse clinical outcomes. The immunological data further reinforces this conclusion. Piglets born to vaccinated sows displayed robust maternally derived antibody responses, as measured by E2-specific blocking ELISA and virus neutralization assays. Vaccination at 44 days of gestation allowed extended time for maternal antibody maturation and colostral antibody concentration, resulting in very high neutralizing titres at birth and during the first week of life. Importantly, these elevated titres do not imply transplacental transmission of antibodies or vaccine virus. In swine, immunoglobulin transfer occurs exclusively through colostrum due to the epitheliochorial structure of the placenta (12). The only ELISA-negative animals were three piglets, identified as numbers 1 and 2 from sow 5 and number 1 from sow 6, which corresponded to stillborn piglets and therefore had not ingested colostrum (Figure 6B). In contrast, all live-born piglets were ELISA-positive from the earliest sampling time point, consistent with rapid and efficient colostrum uptake immediately after birth. Together, these findings reinforce the critical role of early colostrum ingestion in the establishment of passive immunity and indicate that gestational vaccination induced robust maternal humoral responses without evidence of productive fetal infection.

The observation that, in some cases, antibody titres in piglets exceeded those measured in the corresponding sows may appear counterintuitive but can be explained by the dynamics of colostral antibody transfer. Piglets ingest colostrum highly enriched in immunoglobulins during the first hours after birth, leading to rapid absorption and transiently elevated circulating antibody levels. In contrast, antibody levels measured in sows at the same time point reflect serum concentrations rather than the higher antibody content present in colostrum. In addition, individual variability in colostrum intake and absorption efficiency among piglets may further contribute to this pattern. Vaccination at 72 days of gestation also resulted in efficient maternal antibody transfer, although titres showed a physiological decline toward 21–28 days post-birth. This decline is consistent with the estimated half-life of colostrum-derived antibodies in piglets (12) and has practical implications for post-weaning vaccination strategies.

Neutralizing antibody responses deserve particular consideration. Fukai et al. (15), recently proposed an experimentally supported benchmark for maternal protection, demonstrating that piglets with neutralizing titres ≥45 ND50 against the virulent JPN/1/2018 strain were largely protected following challenge, whereas titres <2–5.6 ND50 were non-protective. Although protection was not absolute and variability persisted above this threshold, the study provides a valuable quantitative reference point. When contextualizing the present findings within previously published maternal vaccination studies, clear differences emerge in the magnitude and functional breadth of maternally derived neutralizing antibodies across vaccine platforms. In our study, piglets derived from C-strain-vaccinated sows displayed heterogeneous titres, with only a subset approaching values near the ≥45 ND50 benchmark early in life, followed by a progressive decline before 21–28 days of age. This variability is consistent with previous reports describing heterogeneous maternal antibody levels and their gradual attenuation in piglets born to vaccinated sows (29), as well as ELISA-based studies showing similar trends in maternally derived antibody dynamics (30). In contrast, FlagT4G vaccination, particularly when administered at 44 days of gestation, resulted in a greater proportion of piglets with titres positioned within or above ranges associated with protection in the Fukai model during the early neonatal period (15). Vaccination at 72 days of gestation produced a broader overlap with the ≥45 ND50 benchmark compared with the C-strain group, although titres declined over time. Importantly, the ≥45 ND50 benchmark proposed by Fukai et al. (15) was established against the homologous CSFV JPN/1/2018 strain in a defined experimental challenge setting and should therefore be interpreted within its specific virological context.

A substantial proportion of piglets from FlagT4G-vaccinated sows displayed maternally derived neutralizing titres at or above 1:35–1:40, values historically associated with protection against CSF in experimental settings (31), including against genotype 2 field strains such as Diepholz. These findings are consistent with previous studies showing that marker and subunit vaccines can induce protective maternally derived immunity with variable breadth against heterologous strains (32, 33). Previous studies have demonstrated that maternally derived neutralizing antibodies can confer effective protection against CSFV challenge in piglets, depending on their magnitude and persistence (33, 34), while also potentially interfering with active immunization (14, 35). Importantly, piglets with maternally derived antibodies may still be susceptible to infection when neutralizing titres are insufficient, highlighting the relevance of functional antibody quality rather than total antibody detection alone. In contrast, maternally derived neutralizing responses in piglets born to C-strain-vaccinated sows were lower when vaccination was performed at 72 days of gestation, with most titres remaining below this range. These findings indicate that, even when cross-neutralization against antigenically distinct strains is considered, FlagT4G vaccination results in a quantitatively stronger and functionally broader maternal antibody profile compared with the C-strain under the conditions evaluated. In addition, individual fluctuations in neutralizing antibody titres were observed in some animals over time. These variations may reflect the dynamic nature of the immune response, including temporal changes in circulating antibody levels, as well as the inherent variability of virus neutralization assays such as NPLA, particularly when titres are near the detection threshold. Therefore, isolated fluctuations should be interpreted with caution and do not alter the overall trends observed in the study.

The lack of direct correspondence between total and neutralizing antibody responses has been widely reported. ELISA-based assays detect binding antibodies against structural proteins such as E2 but do not necessarily reflect the capacity of those antibodies to neutralize infectious virus. In contrast, virus neutralization assays provide a direct measure of functional antibody quality, which has consistently been associated with protection against CSFV challenge. Therefore, reliance solely on total E2-specific antibody detection may overestimate protective immunity in vaccinated populations. From a disease-control perspective, vaccines that elicit high binding antibody levels but limited neutralizing activity may reduce clinical severity without fully preventing viral replication or transmission, potentially allowing continued circulation in endemic settings. This distinction underscores the importance of incorporating functional serological assays such as NPLA when comparing vaccine platforms and evaluating long-term control efficiency (5, 12, 22, 25, 26, 36). Comparison with previous studies must be interpreted cautiously, as direct comparability is limited by differences in vaccine platforms, vaccination schedules, challenge strains, assay methodologies, and experimental conditions. Many previous studies have evaluated subunit or marker vaccines requiring multiple doses, whereas the present work focuses on live attenuated vaccines, including the classical C-strain, under defined gestational vaccination conditions. Moreover, not all studies integrate virological monitoring, ELISA-based antibody detection, and functional neutralization assays simultaneously. Therefore, although relevant studies provide important context, comparisons should be made cautiously and within the constraints of each experimental design. Comparison with CP7_E2alf (Suvaxyn® CSF Marker) further contextualizes these observations. Schröder et al. (22) reported peak maternally derived neutralizing titres of approximately 10–40 ND50 following a single vaccination 21 days before farrowing, declining thereafter. These titres remain below the ≥45 ND50 benchmark described by Fukai et al. (15). While differences in vaccine platforms, strains, and experimental conditions must be considered, our results underscore that both vaccine type and gestational timing substantially influence the positioning of maternally derived antibodies relative to experimentally defined protection ranges.

The concept that maternally derived antibodies can interfere with vaccine efficacy has long been recognized (13, 22). More recent analyses further highlight the quantitative and time-dependent nature of maternal antibody interference (14). Within this framework, the gradual decline of neutralizing titres observed after FlagT4G vaccination at 72 days of gestation may align advantageously with the typical weaning vaccination window, approximately 28–35 days of age, balancing early passive protection with reduced interference risk. From a broader perspective, this study addresses a significant gap in the literature, particularly regarding gestational use of the C-strain vaccine, for which detailed data on maternal antibody kinetics and early postnatal detection of vaccine-derived genome remain limited. By directly comparing C-strain and FlagT4G across gestational stages, and by interpreting the results within a quantitative framework anchored to experimentally defined protection benchmarks, our findings contribute practical and mechanistic insight to maternal vaccination strategy design.

Notably, previous studies have demonstrated that FlagT4G is genetically stable, safe for use in pregnant sows, and effective in preventing transplacental transmission under challenge conditions (7, 10, 11). The present study extends these findings by showing that limited vaccine virus RNA detection in piglets is timing-dependent and clinically silent, further supporting the suitability of FlagT4G for gestational use. This study has limitations, including the limited number of sows per experimental group. However, the intensive longitudinal sampling and combined virological and immunological analyses provide a robust basis for identifying timing-dependent trends. In addition, the sow represents the appropriate experimental unit for evaluating maternal vaccination effects, while litter-level analyses provide relevant information on offspring outcomes. Future challenge studies evaluating protective efficacy against homologous and heterologous CSFV strains will be essential to further define the functional implications of the maternal antibody profiles described here.

In conclusion, the timing of sow vaccination with live attenuated CSFV vaccines modulates early vaccine virus RNA detection and the magnitude and kinetics of maternally derived immunity in piglets without compromising clinical health. The integration of quantitative benchmarks such as the ≥45 ND50 threshold (15) and comparative maternal vaccination data strengthens the interpretation of passive protection. While both vaccines demonstrated safety, FlagT4G vaccination, particularly when administered with sufficient time before farrowing, induced more robust and broadly distributed maternal antibody profiles, supporting its strategic value within DIVA-oriented vaccination programs. Effective CSF control therefore depends not only on vaccine choice, but also on the quantitative and functional management of maternally derived immunity. The incorporation of neutralization-based immunological assessment is essential to accurately evaluate protective quality and to support efficient, sustainable vaccination strategies within DIVA-oriented control programs.

## Data Availability

The original contributions presented in the study are included in the article/supplementary material, further inquiries can be directed to the corresponding authors.
